# Oncostatic-Cytoprotective Effect of Melatonin and Other Bioactive Molecules: A Common Target in Mitochondrial Respiration

**DOI:** 10.3390/ijms17030341

**Published:** 2016-03-07

**Authors:** Nicola Pacini, Fabio Borziani

**Affiliations:** Laboratorio Privato di Biochimica F. Pacini, via trabocchetto 10, 89126 Reggio Calabria, Italy; info@fabioborziani.it

**Keywords:** cancer, neurodegenerative diseases, glycolysis, Warburg effect, mitochondria, oxidative phosphorylation, melatonin, indolic compounds, stem cells, apoptosis

## Abstract

For several years, oncostatic and antiproliferative properties, as well as thoses of cell death induction through 5-methoxy-*N*-acetiltryptamine or melatonin treatment, have been known. Paradoxically, its remarkable scavenger, cytoprotective and anti-apoptotic characteristics in neurodegeneration models, such as Alzheimer’s disease and Parkinson’s disease are known too. Analogous results have been confirmed by a large literature to be associated to the use of many other bioactive molecules such as resveratrol, tocopherol derivatives or vitamin E and others. It is interesting to note that the two opposite situations, namely the neoplastic pathology and the neurodegeneration, are characterized by deep alterations of the metabolome, of mitochondrial function and of oxygen consumption, so that the oncostatic and cytoprotective action can find a potential rationalization because of the different metabolic and mitochondrial situations, and in the effect that these molecules exercise on the mitochondrial function. In this review we discuss historical and general aspects of melatonin, relations between cancers and the metabolome and between neurodegeneration and the metabolome, and the possible effects of melatonin and of other bioactive molecules on metabolic and mitochondrial dynamics. Finally, we suggest a common general mechanism as responsible for the oncostatic/cytoprotective effect of melatonin and of other molecules examined.

## 1. Introduction

For over 50 years 5-methoxy-*N*-acetiltriptamine or melatonin (MLT) has been the subject of intense studies and research. Initially, it was considered an endocrine secretion depending on the pineal gland or epiphysis. Then, its *ex novo* synthesis, the enzymes for its formation and its derivatives have been found in organs, tissues and in many different cytotypes [1,2].

Another essential step of studies on MLT is represented by the pharmacological and biomolecular identification and characterization of its receptors, which belong both to the class of G protein-coupled receptors and to that of the ROR/RZR nuclear orphan receptors [3].

Later, many studies in the neuroendocrine and physiological field focused on the relation between MLT and the sleep-wake cycle and regulation of circadian rhythms [4].

Besides these essential aspects, a broad emphasis was given to the well-known reducing and antioxidants properties. MLT was shown to have strong antioxidant properties, even though the precise mechanism has not yet been fully understood. It seems plausible that this involves the oxidation of carbon 2 of the indole nucleus with formation of a carbocationic intermediate and the opening of the indole nucleus with the subsequent formation of kynurenic compounds [5,6].

The relationships between this indolamide and various membrane ion channels are also noteworthy, where MLT is active in interfering on various ionic currents [7,8,9]. Besides a strictly physiological field, the action of ion channel modulator seems to be potentially important also in mediating its known oncostatic properties which will be discussed shortly [10].

Moreover, in scenarios involving the research on MLT we cannot do without mentioning the phylogenetic aspects of this molecule and of the necessary enzymes for its synthesis. MLT is, in fact, widely expressed throughout the world of living organisms, from animals to plants, whether metazoans or non-metazoans [11,12,13,14,15]. This suggests an important ancestral relationship of this indolamide with the physiology and the biochemistry of cells.

Perhaps, among all various aspects of the molecular dynamics inherent in the MLT, the most interesting aspect, and sometimes considered as a debate topic, is represented by its own oncostatic and anti-proliferative properties. For years the physiology of MLT coincided with that one of the pineal gland, and from a strictly historical point of view it is interesting to note that even before the discovery of the MLT, and of the elucidation of the molecular structure by Aaron Bunsen Lerner in 1958 [16], several studies, in the twenties and forties of the last century, showed how the pinealectomy increased the ability of growth of a variety of tumors *in vivo* [17,18]. In addition, between the twenties and fifties of the last century, before the isolation of MLT, various authors reported the beneficial effects of pineal extracts injection in neoplastic patients [19,20,21,22]. Also, in 1957 a complex clinical-epidemiological work documented the relationship between the pineal calcification and breast cancer incidence [23].

In 1963 Rodin confirmed, elucidating techniques, how the pinealectomy and partial pinealectomy enhanced remarkably the growth ability of Walker 256 carcinoma cells in Sprague-Dawley Rats [24]. We owe, then, to El-Domeiri and Das Gupta (1973) the validation through literature that just the lack of MLT was the main factor responsible for the effects of pinealectomy [25].

Starr (1969) reported the first clinical work, performed in the period 1967–1969, on the simultaneous use of intravenous MLT at high doses in combination with adrenocorticotropic hormone (ACTH) in a group of patients affected with sarcomas of soft tissues [26]. It is interesting that Starr associated the ACTH with MLT: while it was known, for some time, that ACTH had an inhibitory action on the secretion of growth hormone (GH), for MLT a similar action had only just been hypothesized. The study of Starr confirmed, both for MLT and for ACTH, an important role in regulating the incretion of GH, and in addition it achieved encouraging results and some permanent remissions for long bouts [26,27].

Studies thereafter confirmed the MLT regulatory role on GH secretion, proving that it could antagonize serotonin at the hypothalamic level [28]. Still with respect to the relationships between MLT and GH, previous pineal studies had promoted this possibility [29,30]. Therefore, the assumption gained ground that MLT has a modulatory effect on the secretion of various hormones including not only GH but also estradiol, lutropin, prolactin, *etc.*, with reference to mammary neoplasm.

Since early in 1973, Burns reported the first study on the possible anti-estrogenic effects of endovenous doses of MLT in women suffering from breast cancer [31]. Other scholars, independently, between the 1960s and the 1970s, utilized MLT in clinical oncology achieving encouraging results [26,32,33]. Since then, several other reports documented a potential use of MLT in clinical oncology.

It is interesting that, just as for the neoplastic diseases, even for the neurodegeneration some clinical trials were conducted, from as early as the end of the 1960s: for example, Antón-Tay *et al.* (1971) reported the beneficial effects of high doses up to 1.2 g/24 h of MLT for intravenous infusion, in people with severe epileptic and neurodegenerative conditions [34], whilst already Papavasiliou *et al.* (1972) described the positive effects of MLT in Parkinson’s disease (PD) [35].

Moreover, starting from the late 1970s, thanks to progress in techniques of biochemistry and molecular biology, as well as in knowledge of cell biology, the oncostatic properties of MLT, of apoptosis induction, of autophagy or sometimes even of death by necrosis and/or aponecrosis, were several times confirmed and clarified in many neoplastic models both *in vitro* and *in vivo* [10,36,37,38,39,40].

## 2. Discussion

Today, with regard to the clinical use of MLT, we should point out how the experiences accumulated so far, sometimes even of phase II, appear interesting and promising, though not yet considered conclusive, unfortunately because of the lack of large-scale randomized studies, the only means, in our opinion, which can settle this long-standing question [41,42]. Another aspect, still much debated in oncology, is represented by the immune-stimulant and immune-modulating actions of MLT, which could exert an indirect beneficial effect through the modulation of several cytokines and autacoids [43]. Although at first glance you might think that MLT is a negative regulator of growth, in numerous studies this substance has been shown to reduce induction of apoptosis, both in neurodegenerative models, such as Alzheimer’s disease (AD), and in models of ischemia/reperfusion [44,45,46].

This apparent dyscrasia, or better, this apparent biphasic aspect on the cell growth and on the regulation of signals of cell death, could represent a key aspect for a better new classification of potentials and actions of this biomolecule.

### 2.1. What Dosage of MLT?

A new, and for us, important point of interest emerged in numerous independent studies, is represented by the dose/dependency, with regard to the selective toxic action of MLT at the expense of neoplastic cells. That is to say, at physiological/subphysiological doses, in some neoplasms but not in all, MLT showed an antiproliferative oncostatic effect, while other neoplasm, insensitive to MLT action at physiological or supraphysiological doses, at high and pharmacological doses show the establishment of MLT-induced cytotoxicity in neoplastic cells [47].

It is in fact documented how the induction of cellular death in cancer cells is mostly active at pharmacological doses; this has been stressed in several cellular lines and *in vivo* [38,39,48,49]. On the contrary, at low doses it can even improve the vitality in many cytotypes, also neoplastic, or appear totally inactive (or active only in some neoplastic forms); it can even exert only an oncostatic effect. In this regard there is interesting evidence in human, in particular with melanoma [50].

Another important figure is the total absence of toxicity, at least as far as we know, also at very high doses, for example in healthy hepatocytes at very high doses, >3 mM until 10 mM, MLT exclusively shows a inhibition of healthy cell proliferation, without expressing cytotoxic effects. If in some large scale clinical trials the results, for example in patients with neoplastic disease, turned out to be discouraging or reduced, an important aspect of constructive critics of these studies could be represented by the dosage and by the pharmacokinetic aspects, on the other hand most *in vitro*/*vivo* studies indicate that only moderately high, or high doses could result in important oncostatic effects.

### 2.2. Neoplasms, Metabolome and Cancer Stem Cells

One of the most interesting and original features in the neoplastic process in biology, is relative to the definition of the cancer stem cell theory. This theory is fundamentally mutating the interpretative models of the cancer process [51,52,53,54,55].

It was highlighted that just the cancer stem cells (CSCs) subpopulation supports cancer growth and is responsible for chemoresistance [56,57,58]. So, in the light of these results and of several other aspects, the stochastic monoclonal paradigm appears today strongly suspect.

Several independent data indicate that neoplastic tissue consists of a heterogeneous population of cells, including the CSCs that preside over self-renewing of the neoplasm. These are associated with various cells being differentiated, albeit in an altered and aberrant form, that make up the vast majority of the neoplastic parenchyma, to which are associated with a role not only of simple support and tropism, numerous stromal cells, such as fibroblasts, which play an extremely important role also in metabolic alterations [51,52,53,54,55]. In parallel, a large number of studies provide a strong revaluation of the link between neoplastic cell and the metabolome [59,60].

The main model holding that the cancer cell relies strongly on glycolysis, with a high lactic acid production, the so-called Warburg effect, has been intensely studied. If Warburg and other scientists assumed that as a cause of the phenomenon there was the incapability of the mitochondrion to utilize oxygen opportunely [61,62], currently we understand how mitochondria of cancer cells possess a high oxygen consumption and a regular activity of the electron transport chain, which is often decoupled both by uncoupling proteins (UCPs) and by other mechanisms [63,64,65,66]. From a large amount of data, it is also clear how the metabolome, in its own adaptable dynamism, may influence many genetic and epigenetic aspects [59,67,68].

There is however a pivotal aspect that can open new perspectives in reference to undifferentiated cell state and metabolomics: totipotent, pluripotent, and the same adult stem cells (ASCs), have a metabolism that, although different in several details, is mostly comparable with the metabolism of the neoplastic cell. In the same way, in the process of reprogramming and induction of pluripotency, there is a metabolic reprogramming, in confirmation of the close interrelation between stem cell and metabolomics, showing and indicating that: for the transition from a differentiated to a dedifferentiated state, such as somatic cells *vs.* induced pluripotent stem cells (iPSCs), we necessarily assist to a metabolic change *vs.* a situation largely comparable to that of the cancer. And similarly an opposite situation in the transition from an undifferentiated state *vs.* a greater differentiation [69,70,71,72,73].

If we acknowledge that the neoplastic process is supported by CSCs, a side population that regulate self-renewal of the neoplastic cells, then a crucial question is portrayed by the source of the CSCs. If the CSCs originate from ASCs, then they have already a metabolism that demands the Warburg effect, which can lead to genetic instability and specific epigenetic patterns [71,74,75].

If, conversely, it is acknowledged that the CSCs originate from a somatic cell reprogramming process, then these metabolic features represent a condition not subject to the cancer transformation, but necessary for the reprogramming. Conversely, independently of the path that leads to the origin of CSCs, an altered metabolic state can largely affect the anomalous self-renewing processes and the anomalous differentiation of the neoplastic parenchyma [76,77,78]. Hence, based on an accurate analysis of the literature we created a model that describes how the onset of neoplastic process is linked to the simultaneous alteration of epigenetic patterns, of genetic stability and of the metabolome [59].

Finally, it should be remembered that the metabolic state is important in regulating the metastatic processes and it could depict a drive towards the epithelium-mesenchymal transition (EMT) [79,80]. Recent validations seem to corroborate this perspective on the EMT [81,82]. Many of our concepts and the very idea of a link between metabolomics, epigenomics and genomics on the one hand, and self-renewal and CSCs on the other, have been topic of discussion for many authors [83,84,85,86,87,88,89,90,91,92]. Moreover, several recent studies seem to confirm and to share many aspects of our model, including the importance of the relationship between ATP/ADP and EMT [81,83,93,94,95,96].

Some important authors interpreted our model as a reinterpretation of the concepts expressed by Warburg more than 100 years ago [97]. This interpretation is however erroneous; indeed, in our model, epigenetic, genetic and metabolic aspects are considered according to an integrated point of view; we also affirm that the metabolic alterations, typical of the neoplastic process, represent a necessary, but not a sufficient, concomitant cause in the genesis of tumors. That is a cooperative model, which states unambiguously that the triple simultaneous cooperative alteration among genetic, epigenetic and metabolic systems, drives the genesis of the neoplastic process. Also in our model, the mitochondrion is regarded as one of the main creators of the metabolic alterations and of the modifiers of transcription, not simply considering the absence of respiration, as postulated by the primary hypothesis of Warburg, but the more complex reality emerging from numerous studies. We have therefore also considered the UCP-mediated decoupling, in addition to multiple other aspects. Starting from the link between a proper aerobic metabolism and cellular differentiation, we ultimately present a model according to which the main metabolic alterations, affecting many epigenetic mechanisms and remodeling of nucleic acids (gene stability), are found on the stem segment of the neoplastic bulk. This model, based on the cooperative alteration of three systems, shows, particularly, an alteration of the stem compartment, and not of each neoplastic cell [92,98,99,100,101,102,103].

We believe that the main cause of these metabolic alterations is the loss of the correct respiratory mechanism, however this does not recall the original Warburg paradigm about the total absence of respiration or about the inefficiency of the rate of O_2_ consumption; but we believe that there is, as an essential mechanism, the decoupling and the alterations of respiratory complexes. Recent studies appear to confirm a vision of the problem in this regard, and confirm the close link between stem cells and metabolomics, and an increasing number of reports confirm a pivotal role of the metabolome in the genesis of tumors, and a close link between the stem state and the metabolome [83,84,85,86,87,88,89,90,91,92].

### 2.3. Neoplasms and Mitochondria

In studies *in vitro*, and *in vivo*, in anatomical and pathological findings, there is much evidence of significant alterations in the quantity of neoplastic mitochondria, in their morphology, in their biogenesis, in their function, as well as in all neoplastic mithocondrial respiratory complexes in comparison to healthy cells. This results in a strong reduction of ATP production through oxidative phosphorylation in favor of processes of phosphorylation at the substrate level, mainly both through glycolysis and glutamine metabolism [63,64,65,104,105,106,107,108].

As mentioned above, historically Warburg hypothesized in 1956 that originally the neoplastic process showed the inability on the part of the respiratory cytochromes to express a correct oxygen reduction, in other words, to use it properly [62]. However, this hypothesis had previously been contested by Lynen, who experimentally verified how often the consumption of oxygen and its reduction were still present in neoplastic cells [109]. About this we should also mention the memorable dispute between Warburg and Weinhaus, who also had observed a high oxygen consumption [110].

It has been proven for many years now, and confirmed on several occasions, that mitochondria of neoplastic cells actually have a strong decoupling of the oxidative phosphorylation, that is of oxidation of phosphorylation. This is mediated by various decoupling proteins, particularly UCP2, but also by factors not yet fully identified. It equally acts as a decoupling condition, a strong transport of electrons through the electron transport chain, and in many findings, also an excessive oxygen consumption, compared with untransformed cells. On the other hand, the dry measure of oxygen consumption is not a good method for determining the proper production of ATP through the mitochondrial pathway [59,63,64,65,107,111,112]: it is now clear that the mitochondrion, regardless of the production function of ATP, that is of the oxidative phosphorylation, performs several key functions, including the essential role of pO_2_ sensor and regulator. In fact, it can generate local hypoxia and it can modulate the pO_2_, facts that could have also important implications for the survival of neoplastic cells. Moreover, the oxygen consumption and the generation of reactive oxygen species (ROS), and in particular hydrogen peroxide, play numerous roles in many pathways [113,114,115,116,117,118].

In neoplastic cells *in vitro*, *in vivo*, and in histopathological findings in clinical medicine, numerous variations in expression, in proper functioning and in the activity of all the main respiratory complexes, have been extensively documented. In particular, in many cancers there is a downregulation of the expression and activity of complex I, or the complex NADH-CoQ oxidoreductase, of complex II or succinate CoQ reductase, as well as of complex V or ATP synthase, while the situation is more heterogeneous for complex III or the complex of the coenzyme Q-cytochrome c reductase, which often, but not always, seems to be overexpressed in many neoplastic cells. Also this was confirmed *in vitro* and *in vivo* and in clinical findings [104,119].

In a refined study on genomics and proteomics Owens *et al.* showed that in a large number of human mammary tumors a strong decrease in expression of complexes I, II, IV and a high expression of some components of complex III is evident [120]. This structure is a protein complex of 248 kDa which includes 11 subunits of 10 gene products. The 11th subunit comprises the mitochondrial target sequence of the Rieske protein (RISP), and an iron-sulfur center essential for the electron transfer to cytochrome c. This gene product RISP is divided and inserted in the transmembrane domain of complex III. The overall results by Owens *et al.* indicate a strong and constant overexpression of ubiquinol-cytochrome c reductase, the product that encodes the protein of RISP, along with an overexpression of cytochrome b-c1 complex subunit 6 zipper sequence. These gene products are altered and overexpressed in a variety of malignancies, so these scholars have evaluated *in vitro*, in MCF7 cells and 143B osteosarcoma cells, effects of knock-down of RISP. This leads to a strong decrease in invasiveness and a reduction of the expression of NADPH oxidase (NOX), of which we have previously discussed, in particular the reduction of the expression of NOX2, NOX3, NOX4, NOX5, leaving unchanged the expression of NOX1. It is also important to consider that if on the one hand the invasiveness decreases, on the other hand the knock-down of RISP gives remarkable resistance to apoptosis; interestingly, even triple negative breast cancer cells had the same structure. Consistent with this work, many other reports suggest a pivotal role in the modulation of complex III and regulation of apoptosis.

At the level of this delicate complex, electrons from the reduced ubiquinone or ubiquinol are released. Ubiquinone plays a crucial role in the transport of electrons, receiving electrons from complex I, or the complex of NADH-CoQ oxidoreductase, from complex II or the complex of the succinate-CoQ reductase, from glycerol-3-phosphate dehydrogenase and other electron transfer flavoproteins. It is remarkable that exactly complex III, that, as mentioned above, is highly overexpressed in human cancers, and in particular the metabolism of ubiquinone that takes place therein, plays an essential role in regulating the expression of uncoupling proteins [121,122], to emphasize a possible mutual reinforcement, since, as already stressed, UCPs are abundantly expressed in cancer cells where they play an essential role [59,63,64,65,107,111,112]. On the other hand, just the expression of UCP is fundamental in mediating also a response to the excess of glycolysis [121,122].

A similar situation was also documented for complex IV or cytochrome c oxidoreductase, which appears overexpressed in many pathologic findings and in many *in vitro* studies. This structure consists of 13 subunits, three of which are encoded by mtDNA and 10 at the nuclear level. The synthesis of two nuclear subunits, important for the function of the complex, seems to be under the direct control of the pO_2_: these subunits are the Vα and Vβ. Also, mutations in the mtDNA coding complex IV and their upregulation are associated with a high increase in malignancy and with a worse prognosis [117,123].

Complex III and its own activity, in coordination with complex I, also seem to play an essential role in the chemoreception of pO_2_ and in the synthesis of the factor inducible from hypoxia or hypoxia-inducible factor (HIF-1α), that, as we know, is often invoked as a cause or contributory cause in the genesis of the Warburg effect. Several reports show a more complex and heterogeneous situation: the activity of these respiratory complexes appears not only essential in chemoreception of pO_2_, but also in the induction of a state of hypoxia related to the content of mtDNA. As shown by Prior S. *et al.*, a deregulated and abnormal mitochondrial function can induce a strong hypoxic state, regardless of the pO_2_ in various models of breast and prostate cancer, inducing and maintaining through this mechanism the synthesis and stabilization of HIF-1α, a phenomenon which is in accordance with other studies and previous data, and that, according to the group of Higuchi, is related to the concentration of the mtDNA [124]. However, even by means of these mechanisms the mitochondrion exerts an essential control on the intracellular oxygen pressure, on the synthesis and stabilization of factor HIF-1 and the genesis of the Warburg effect [113,114,115,116]. The evidence suggests a complex and heterogeneous situation in which the mitochondrion and the activity of the respiratory complexes work, not only as acceptors and reducing agents of oxygen, but also as a complex and delicate rheostat that regulates pO_2_.

In recent years, numerous and independent validations also highlighted the pivotal role played by complex I and III in physiological and pathophysiological ROS production, and in particular of H_2_O_2_, a molecule that like NO, could play an important role as a cellular messenger and be involved in the processes of differentiation, apoptosis and in various physiological and physiopathological aspects. Similarly H_2_O_2_ seems to be involved in the most important pathways associated with life-span and the cell cycle, such as the mammalian target of rapamycin mTOR pathway [125,126].

As long known, the formation of superoxide anion is a direct function of pO_2_ and of the amount of oxidizable substrates, according to the following kinetic formula:

$$\frac{\left(d[superoxide]\right)}{dt}=k[O_{2}][AH]$$

in which, indeed, there is a direct proportional relationship among oxygen pressure (O_2_), presence of reducing substrates (AH such as NADH), and ROS production, indicating how enzymatic processes do not participate in the formation of the superoxide anion O_2_^−.^. In the presence of a stochastic process it was assumed that normally 1%–2% of the oxygen consumed is reduced to superoxide anion [127].

On the other hand the very formation of ROS and the activity of manganese-dependent superoxide dismutase MnSOD represents a central link with the Warburg effect, and the genesis of many metabolic alterations. Just the delicate balance in the activity of MnSOD, and in the ROS formation, regulate many metabolic aspects: for example, as shown by (Xu *et al.*), in MnSOD-heterozygous knockout (Sod2^+/^^−^), mice, there is an increase in ROS formation that leads to the activation of the peroxisome proliferator-activated receptor alpha (PPAR-α), which induces the expression of uncoupling proteins (UCPs), which ultimately, through the PI3K/Akt/mTOR pathway, stimulates aerobic glycolysis [128]. Paradoxically, Hart P.C. *et al.* in breast tumors *in vitro* and *in vivo*, showed that it is just the upregulation of MnSOD, through an overproduction of H_2_O_2_, which modulates the action of AMP-activated kinase, stimulates the aerobic glycolysis and the so-called Warburg effect [129]. Actually, by a closer and more detailed analysis in literature, we see that just the level of ROS, of hypoxia generated and regulated at mitochondrial level, regulates and modulates the intensity of aerobic glycolysis [130,131,132]. In other words there is a delicate balance among O_2_ consumption, ROS production, activity of respiratory complexes and genesis of Warburg effect.

Paradoxically, multiple and independent studies suggest that under conditions of hypoxia, or of reduced electron transport, the electron transport chain generates an overproduction of ROS, and in particular, H_2_O_2_, which seems to have an important role in the synthesis of HIF-1. In order to explain this paradoxical effect we have to consider the model proposed by Mitchell (1975) for the “Q-cycle”: the inhibition of the site Qi, for example with antimycin A, is associated with a strong production of ROS [133]. In fact, blocking or perturbing the transfer of electrons from the semiquinone radical to Eme 555, we can see strong radical cascades, as the semiquinone radical may react with other cellular structures. At this level, the production of ROS is extremely harmful for the mitochondrial structures, but also for the whole cell, where it can come out through the voltage-dependent anion channel [134].

### 2.4. Apoptosis, Aponecrosis and Necrosis: A Common Denominator

It is now almost 43 years since Kerr *et al.* coined, in a historical article, the term apoptosis to describe the morphology of a defined program of cellular death, as opposed to the classical morphological picture of death by necrosis [135]. Since then, numerous biochemical and functional studies have clarified and elucidated many mechanisms governing the fine regulation of apoptosis [136,137,138,139].

Numerous essays and monographs define apoptosis as a synonym of “programmed cellular death”. This phenomenon is not yet comparable to the apoptotic process in its entirety, that is to the programmed cellular death that was already well known to the morphologists of the nineteenth century, who had well studied the reabsorption of embryonic anlages during homogenesis and the physiological endometrial remodeling in menstrual periods.

For example, in the nematode *Caenorhabditis elegans*, which during its development loses 131 of its 1090 somatic cells, specific inhibitors of caspases, that block apoptosis, do not prevent cellular death of these cells, which, on the contrary, undergo death by necrosis. These and other findings suggest that apoptosis represents an adjusted mechanism of cellular death, but that it does not mean programmed cellular death. This type of cellular death has been defined as “cellular suicide” and “programmed cell death”. Paradoxically, in the early 1990s, it was identified in unicellular organisms such as the yeast *Saccharomyces cerevisiae* [140]. Numerous studies documented the existence of the intrinsic or mitochondrial pathway of apoptosis, or the process mediated by the release of cytochrome c by the activation of the apoptosome. It was also well confirmed that mainly in the formation of a colony, *Saccharomyces cerevisiae* has a markedly glycolytic phenotype, at a low oxidative phosphorylation and low ATP reserves. This is associated with a low level of apoptosis, where, once the plateau of a colony is reached, oxidative phosphorylation and the reduction of metabolism through the glycolytic pathway, with a percentage recovery greater than the apoptotic process is resumed [141]. In this sense we can speak perhaps of homeostasis in the number of the elements of a population, or the problem of the common good. Both apoptosis and necrosis, however, have many things in common represented by the loss of mitochondrial homeostasis, by the efflux K^+^ ions across the cellular membrane, by various perturbations of ionic homeostasis and of mitochondrial permeability [142,143].

So, if on the one hand apoptosis has been historically opposed to necrosis, a process organized and finely regulated, on the other hand today much evidence suggests that the necrosis itself, according to stimuli that cause it, as well as the intensity of these, can partly represent a phenomenon much less chaotic than what we have believed to date. Since numerous intermediate situations have been described, ranging from apoptosis to necrosis and including aponecrosis, it is preferable to speak of “cellular death”, a more inclusive term.

Apoptosis is a finely regulated process and controlled by various proteins: in the intrinsic pathway of apoptosis a pivotal role is played by the release of cytochrome c from the inner mitochondrial membrane to the cytoplasm, where binding to proteins APAF-1 and to caspase-9, it forms the apoptosome, which, through the activity of ATP hydrolysis, activates the apoptotic process, with the activation of caspase-3. From the inner mitochondrial membrane other proapoptotic factors are also released, such as apoptosis inducing factor, a nuclear DNAase and the protein diablo, which inhibits other antiapoptotic cytoplasmic factors. The regulation of these processes is tightly controlled by the genes of the BCL-2 family, and by their homologs. Initially, the founder of this BCL-2 family was identified as an anti-apoptotic factor in leukemia and lymphoma cells. Subsequent studies showed also other members of this family, with a high sequence homology with proapoptotic activity, such as BAX and BAK. BCL-2 expresses its own anti-apoptotic function by forming heterodimers of BCL-2-BAX, consisting of the interaction of N terminals BH1, BH2, and BH4. The link between BCL-2 and BAX prevents the formation of homodimers of BAX, that activate the complex of mitochondrial permeability and the release of cytochrome c by facilitating the initiation of the apoptotic process. There is therefore a delicate balance in the relationship between BAX and BCL-2. In the formation of the mitochondrial permeability pore a pivotal role is also played by the voltage-dependent anion channel and the adenine nucleotide translocator, as well as the rising of levels of [Ca^2+^]_i_. BCL-2 is found primarily in the intermembrane mitochondrial space, in the nucleus and endoplasmic reticulum [138,144,145,146,147].

Several reports have suggested an activity of BCL-2 in the regulation of oxide-reductive homeostasis and as a possible free radical scavenger; however, more detailed studies showed a dualistic role of BCL-2 in the regulation of ROS levels. More precisely BCL-2, in absence of oxidative stress, seems to bind the Vα subunits of the cytochrome c oxidoreductase or complex IV and to facilitate its transfer to the inner membrane, as well as the proper assembling of the complex, causing an increase of ROS and of oxigen consumption, effects in part mediated by the interaction of BCL-2 with the Rac1 GTPase of the Rho-kinase family. The resulting generation of ROS has then important effects at various levels. Inversely, in basal conditions of oxidative stress, BCL-2 seems to inhibit the transfer of the COX Vα complex and reduce its function. It also seems to be extensively involved in moving abundant reserves of reduced glutathione to mitochondrial structures. Another important fact is represented by the link among respiratory activity, considered as respiratory consumption, activity of all the respiratory complexes, and expression of BCL-2, which is largely influenced by these parameters: in fact, in many cellular models the blocking or perturbation of the electron transport chain, or the reduction of its activity, is associated with a reduced expression of BCL-2, increased expression of BAX and cellular death [117,123].

### 2.5. Melatonin and Mitochondria: A Developmental Liaison?

Various evidence suggests that the mitochondrion is the main focus of the action of MLT: enzymes *N*-acetyltransferase and hydroxyindole-*O*-methyltransferase are present in the mitochondrion, and this essential sub-cellular organelle is the site of synthesis of MLT itself, aspects of which can also be found in chloroplasts. It was thus suggested, in consideration of its reducing characteristics, that MLT is ubiquitous and present in these organelles as a natural non-enzymatic defense mechanism in respect of oxygen-mediated toxicity [148].

A variety of independent studies have now clearly shown that MLT, in *in vitro* and *in vivo* models of neurodegeneration, such as AD and PD, as well as in models of ischemia-reperfusion or in liver damage induced by various agents, decreases the rate of apoptosis, to prevent the formation of the mitochondrial transition pore, to stabilize and maintain the mitochondrial membrane potential and prevent the release of cytochrome c [44,149,150,151].

On the contrary, in numerous neoplastic models it induced cellular death, in a dose-dependent way, often showing not only morphological structures of apoptosis, but also of aponecrosis and violent ROS-mediated cellular death (necrosis), accompanied by destruction of mitochondrial structures and by the loss of any respiratory process. Analyzing all the literature, this effect appears also dose dependent [10,48,49,152,153,154,155,156,157]. This discrepancy, about the action of MLT on untransformed cells, compared with neoplastic ones, could represent the true fundamental matter, with regard to its action and to links with the mitochondrion.

In isolated mitochondria, in cells and *in vivo* models, MLT acts directly on the enzymatic activity of respiratory complexes I and IV, greatly improving their function, and antagonizing its toxicity induced by rotenone (inhibitor of complex I) and 1-methyl-4-phenylpyridinium. Globally, MLT exerts a stabilizing action of the mitochondrial membrane potential, decreasing the consumption of oxygen, reducing phase 3 mitochondrial respiration, modulating the respiratory control index (ICR = $\frac{V_{3}}{V_{4}}$), and interfering also on entry of reducing substrates in the Krebs cycle [158,159,160,161,162,163,164,165,166]. Still with regard to the relationship between MLT and respiratory complexes it should be noted how it inhibited, in various models, the toxicity induced by doxorubicin and other anthraquinones, which, as we know, perturb the mitochondrial chain complex I, functioning as electron donors [167].

In brief, an extensive literature provides evidence that MLT improves mitochondrial function, stabilizes the electron transfer complexes I and IV and prevents electron leakage [168]. In particular, its effect, particularly in models of ischemia-reperfusion, seems to be mediated by a decrease in the consumption of oxygen, a prevention of electron leakage and an overall improvement in the efficiency of oxidative phosphorylation [150,169]. Also, MLT could participate directly in the reduction of NAD^+^, increasing the stock of NADH and the electron transfer [170], as well as it could participate in the gene modulation of the expression of various respiratory complexes [171]. Paradoxically, although it improved the toxicity induced by excessive ROS and by anthraquinone-chemotherapy, such as doxorubicin, in neoplastic cells it seems to potentiate the cytotoxicity of ROS and doxorubicin [154,172], as already expressed inducing cellular death through the formation of ROS.

Evidence suggested a direct link of MLT with respiratory complexes: some of its effects on mitochondria are not in fact receptor-mediated. Its own direct link with the cytochrome c oxidoreductase or complex IV, which *in vitro* oxidizes MLT with formation of 2-hydroxy-MLT was also suggested. From this and other evidence described above, a direct action of MLT against respiratory complexes and mitochondrial structures is evident [168,173].

Reports suggested a special relation between MLT and complex III of the respiratory chain: in this regard, the studies effected by Zhang *et al.* (2011), Zhang *et al.* (2011) and Fu *et al.* (2013) were very interesting, showing that in glomerular mesangial cells MLT, at pharmacological doses, as well as already documented in other studies, induces a large production of ROS and that the inhibition of complex I of the respiratory chain via rodanone has no effect on the production of ROS induced by MLT [174,175,176]. The inhibition by means of myxothiazol, which binds the site Qo, reduces this effect, while the antimycin A, which is a powerful inhibitor which binds the side Qi or Qn of complex III, inhibited completely the formation of ROS induced by MLT, data also confirmed in leukemic cells by Perdomo *et al.* (2013), where the ROS-induced cell death was abolished by Antimycin A [157].

Another interesting aspect pointed out by the study of these researchers is that during the generation of ROS, MLT does not seem to actively participate in the oxido-reductive process, and it is not even subject to ossidation, globally suggesting that MLT may bind the site Qi of complex III, or site of antimycin A, and can perform some modulating activity, or even could be defined as an allosteric modulator of the enzyme [174,175,176]. Subsequently, the same researchers developed a fluorescence method for the evaluation of the enzymatic complex III, based on the link between MLT and the same complex III [176]. It is interesting to observe that the presence of the methoxy group in position 5′ of the indole ring is essential, as well as its aromaticity, where hydrogenation in 3′ completely inactivates this effect. Also the ethylamidic chain is important, where the indole derivative 5′ methylated, devoid of the chain in 3′, is active but much less active in inducing this effect.

The measurement of activity of the electron transport chain, or more precisely the oxidation of 2′,7′-dichlorodihydrofluorescein, promoted by MLT and strongly inhibited by antimycin A, was much more evident in cancer cells than in non-neoplastic cells, confirming what was already expressed in the literature on the relationship between electron transport and oxygen consumption in neoplastic cells.

According to Zhang *et al.* (2011), in an *in vivo* model of renal injury induced by diabetes in mice db/db, where in kidney cells we would have been expected a strong production of ROS induced melatonin, surprisingly MLT has not induced ROS production [175]. A careful analysis revealed that this effect was caused by the high downregulation of complexes III and I, suggesting that MLT action is not unique and constant, but is rather in relation to metabolic conditions, such as the expression of various respiratory complexes, the speed of electron flow through the electron transport chain and oxygen consumption. In this sense it must also be considered that MLT, in prolactinoma cells, under the stimulating action of 17β-estradiol, which is known to upregulate the expression of all the respiratory complexes and particularly of III, showed a marked dose-dependent inhibition, not only on complex III but also I and IV, with greater effects on III and an induction of ROS mediated cellular death, with loss of mitochondrial function [177]. In this regard, also the confirming results reported *in vivo* by Acuña-Castroviejo *et al.* (2012) are significant [178].

Analyzing the activity of other indole compounds and their Structure Activity Relationship (SAR) it is not surprisingly a possible action of MLT on complex III: in particular, Tutton and Barkla (1977) demonstrated, in a model of colon cancer, the cytotoxicity induced by an indole derivative, the 5,6-dihydroxytryptamine (5,6-DHT) [179]. This indole molecule induces in this cell model a framework of necrosis, and the destruction of mitochondrial structures, without inducing an excessive toxicity in healthy epithelial cells.

This substance, which seems to have remarkable structural similarities with MLT, and yet more with 6-hydroxymelatonin (6-OHM), the main MLT catabolic derived, 5,6-DHT, has been extensively studied and abandoned because at high doses it exerts a neurotoxic activity. However, a great deal of studies *in vitro*, *in vivo* and on isolated mitochondria, through polarographic techniques, have shown that 5,6-DHT induces an increased production of ROS mediated through a strong stimulation of complex III: this effect is experimentally reversible with antimycin A and it is connected to the binding of this indole molecule with the Qi site of complex III [180,181,182].

In the classical model MCF-7, Shellard S.A. *et al.* (1989) tested the efficacy and potency in inducing cell death of 5,6-DHT, of 6-OHM and of MLT, highlighting an increasing toxicity in the order MLT < 6-OHM < 5,6-DHT [183]. A strong pro-oxidant and cytotoxic action with formation of H_2_O_2_ by 6-OHM, similarly to 5,6-DHT, has also been highlighted in human leukemia cells HL-60, in which exactly 6-OHM induces cellular death and DNA damage, while in HP100 leukemia cells, resistant to H_2_O_2_-induced cytotoxicity, it is without effect [184].

6-OHM is highly unstable and ultimately the isoform CYP1A2 of the cytochrome P450, which exactly catalyzes hydroxylation at position 6′ of MLT, is strongly upregulated in many human cancers, such as breast cancer and not only, where you can have a rate of expression greater than 200-fold compared to healthy tissue [185,186]. At the same time, the formation of 5,6-dihydroxy-*N*-acetyltryptamine and other derivatives structurally similar, such as 5-methoxytryptamine and *N*-acetylserotonin (NAS), is quite possible and in some ways evident [187,188,189,190,191,192,193]. On the one hand it strongly suggests an effect like-5,6-DHT for 6-OHM and MLT; on the other hand, the formation of 5,6-dihydroxy-*N*-acetyltryptamine is also likely. This suggests that minor fractions of these metabolites may have an important role.

**Figure 1:**
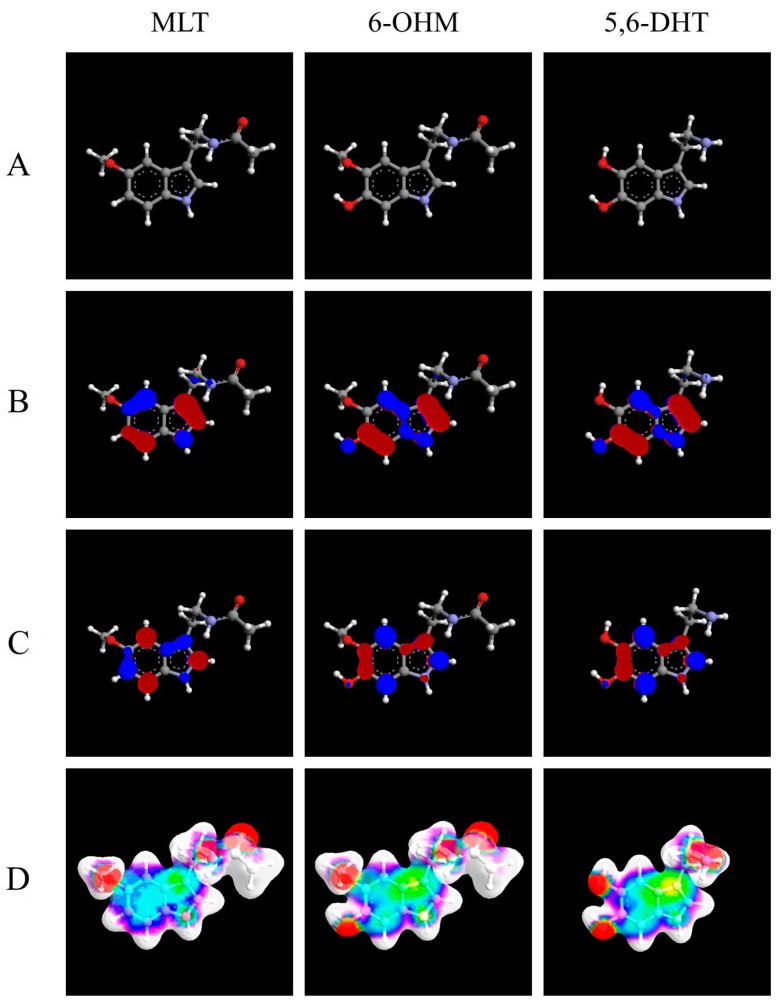
3D molecular structures of melatonin (MLT), of 6-hydroxymelatonin (6-OHM), and of 5,6-dihydroxytryptamine (5,6-DHT). (**A**) 3D molecular structure to note the distances between 2 oxygen atoms of 6-OHM and 2 phenolic hydroxyls of 5,6-DHT, respectively 2.77 and 2.82 Ångström (Å); (**B**) Frontier orbitals: HUMO (highest occupied molecular orbital); (**C**) Frontier orbitals: LUMO (lowest unoccupied molecular orbital); (**D**) Electrostatic Potential Surfaces (ESP). The geometry of the systems has been optimized considering the semi-empirical molecular orbital theory at the level of AM1 (Austin Model 1) and the electronic properties of the systems have been calculated by *ab initio* Restricted Hartree-Fock). ArgusLab 4.0.1 software (Mark Thompson and Planaria Software LLC, 2004) was used.

In Figure 1, Table 1 and Table 2 some common characteristics of MLT, of 6-OHM and 5,6-DHT showing how these molecules exhibit strong structural and functional similarities, are highlighted.

The data reported are in agreement with what has already been observed by Erkoç *et al.* (2002) regarding MLT and 6-OHM [194].

### 2.6. MLT and HIF-1α, Hypoxia-Reoxygenation

Certainly MLT may affect important relationships between metabolomics and neoplasms through multiple mechanisms such as a downregulation of hypoxia inducible factor HIF-1α, a fact now documented for a long time and through independent studies [195,196,197,198,199]. On the other hand, the synthesis, the stabilization and activation of the factor HIF-1α are regulated through the formation of ROS and particularly of H_2_O_2_. It has often been hypothesized that just one of these non-specific scavenger activities can play a very important role in the regulation of the synthesis and stabilization of the HIF-1α factor. However, the dynamics of the relationship between HIF-1α and MLT may be more complex and articulated, as the regulation of HIF-1α is under the control of complex III [113,114,115,116].

On the other hand there are close relations between pO_2_ and MLT: for example, in a line of human syncytiotrophoblasts, MLT turned out to be useful and active through various pathways in modulating and antagonizing the damage from hypoxia-reoxygenation. However, in the transformed counterpart, namely in the choriocarcinoma cell line, MLT induces apoptosis mediated by the induction of the transition pore and by the loss of mitochondrial function [200]. Possible relations between MLT and pO_2_ were also investigated and analyzed in invertebrates, such as for example in *Triturus carnifex*, where it seems to mediate a prompt response to hypoxia and anoxia through the synthesis and catabolism of melanin in liver Kupffer cells [201,202]. The multiple actions of MLT on the relationship between pO_2_ and HIF-1α may be mediated not only through direct mechanisms involving the respiratory complexes, but to a lesser extent also by indirect mechanisms on the regulation of many hormones such as thyroid hormone [203].

### 2.7. A Common Set of Mechanisms for Different Molecules?

The paradox of MLT, namely a strong cytoprotective action in AD, PD models, in models of ischemia-reperfusion and many others, and conversely a strong cytotoxic and antiproliferative activity in cancer cells, is common in many other molecules. We just mention a few examples: 3,5,4′-trihydroxy-trans-stilbene, or resveratrol, green tea catechins, derivatives of vitamin E as α-tocopheryl succinate, capsaicin and many other substances for which a marked effect on the activity of respiratory complexes was highlighted, which is associated with a paradoxical scavenger action in models of neurodegeneration and with a high formation of ROS and H_2_O_2_ in neoplastic cells [204,205,206,207,208].

It is well known that in many models of neurodegeneration, such as in multiple sclerosis (MS), PD, AD, numerous metabolic alterations have been well documented and described, sometimes different and opposite, but which have a common thread, characterized by reduced activity of mitochondrial respiratory complexes. For example, in MS a strong association between polymorphisms of complex I and its alteration with reduced activity has been well documented. Similarly in PD, the close link between complex I, its activities and the genesis of the disease in experimental models, such as those induced by 1-methyl-4-phenylpyridinium, is well documented. In AD, although it has been described a high respiratory activity and a greater degree of oxidative phosphorylation, numerous alterations involving the hypoactivity of respiratory complexes, in particular of I, III and IV of the respiratory chain complex, have been described. Moreover, numerous data show a close link among reduced ATP/ADP ratio, decreased activity of respiratory complexes and cellular death by apoptosis induction [209,210,211,212,213,214,215].

Recently, Bobba *et al.* (2015), showed that in various models of AD, and in cerebellar granule cells, an upregulation of the glycolytic pathway was associated to marked drop in the early stages of apoptosis [216]. The so-called “numbness of mitochondrial activity” seems to be an essential stage of the apoptotic process.

As mentioned above the group of Low *et al.* documented in a large number of studies the close relationship between complex IV, its activity and BCL-2: in one of their studies they showed that the effect of induction of ROS by resveratrol, is partly antagonized by the overexpression of BCL-2 [123]. Indeed, various studies already confirmed by other literature, demonstrated that BCL-2 can increase or decrease the function of COX-IV, exerting a dual role as a pro-oxidant and antioxidant respectively: these actions are mediated by BCL-2 and by their interactions with the Vα subunit of the complex IV [117,123].

In brief, we can conclude that:

(a) the activity of respiratory complexes is diminished in many models of neurodegeneration;
(b) likewise, the activity of respiratory complexes is strongly upregulated in neoplastic cells, which show UCP-mediated uncoupling and, at the same time, high respiratory consumption;
(c) in response to a rise in the activity of complex IV and III, many neoplastic cells respond through the reduction of the enzymatic activity of these complexes in order to avoid catastrophic free radical events.

In this context, the paradoxical action of MLT, able to induce cellular death in cancer cells and cytoprotection in models of neurodegeneration, is quite appropriate. This molecule, in fact, stimulates the activity of respiratory complexes I, II, and IV, and has a marked effect on complex III, thus being able to achieve a strong perturbation of the electron transport chain in neoplastic cells, also preventing the braking action of BCL-2 and overstimulating an already metastable cellular system, characterized by a high electron flow through the electron transport chain, high oxygen consumption, UCP-mediated uncoupling and high sensitivity to ROS.

Conversely, a diametrically opposed but paradoxically similar situation, namely a high glycolytic flux but a low oxygen consumption and reduced expression of UCP-2 [217,218,219,220], is frequently observed in neurodegeneration and in many other situations of cell damage. MLT can correct exactly this hypofunction of respiratory complexes, increase ATP reserves, intervene through scavenger mechanisms of free radicals and modulate oxygen consumption, a context which is even more reasonable if we think of the opposing effects of many molecules such as derivatives of ubiquinone and vitamin E in two opposite pathophysiological models of cancer and neurodegeneration [87,92,100,102,103,221,222,223,224,225,226,227,228,229,230].

Of course, for MLT, as for the other molecules described above, there are also many other mechanisms of action, both receptor-mediated and of adjustment on other systems: for example, MLT has many receptor-mediated actions, a number of important effects on the release of several hormones and autacoids such as NO. In fact, just through the modulation of the levels of NO, MLT could regulate numerous aspects of glucose metabolism [231].

Also, in light of the essential bond existing between stem cell compartments of the CSCs and neoplasms, this indole molecule may also have a role in the regulation of pathways related to Notch, which also plays an important role in the maintenance of self-renewal. However, these mechanisms of the electron transport chain, in view of the recent findings on the metabolome, likely represent the essential core of the effects of these molecules. Finally, in the case of MLT, a particular situation occurs: this indolamine generates *in vivo* highly active derivatives such as 6-OHM, the *N*-acetilserotonine and, potentially, also the generation of 5,6-DHT is possible. In brief, MLT is part of a highly dynamic context that might be considered an interrelated system of indole compounds. It also appears more important if we analyze the phylogenetic context of MLT and of enzymes necessary for their own synthesis.

## 3. Conclusions

As described above, there are close relationships between metabolomics and cancer, and between metabolomics and neurodegenerative diseases; this phenomenon is so evident that the metabolome and its alterations can be defined as a common factor of two opposing situations, respectively characterized by an excessive and abnormal cellular proliferation and, on the contrary, in neurodegeneration, by a high rate of cell death. As crucial turning point of these two opposite situations, mitochondrion and cellular respiration play a decisive key role. Although with some general exceptions, it is, in fact, demonstrated that in neoplastic cells aerobic glycolysis prevails, supported, in many cases, by a high cellular respiration (high consumption of oxygen), decoupled by phosphorylation. High levels of ROS that have been produced support various cellular signaling that stimulate the proliferation and invasiveness and confer resistance to apoptosis, while in the opposite situation regarding the major neurodegenerative diseases we see a prevalence of glycolysis, associated with low cell breathing, with reduced energy stocks and facilitation of the apoptotic process.

Many mitochondriotropic substances stimulating respiratory processes (mitocans) are associated with scavenger and anti-apoptotic activity in models of neurodegeneration such as PD and AD, while they activate cellular death both apoptotic and for necrosis or aponecrosis in many neoplastic models both *in vivo* and *in vitro*, through the genesis of ROS. These both similar and different effects can lead to a possible mechanistic explanation, since the substances stimulating the activity of the respiratory complexes and opposing the excess of glycolysis, which precedes apoptosis in neurodegeneration, can slow or re-invert this mechanism of cellular death, while the same stimulation in cancer cells, which show, already at basal levels, a high oxygen consumption, a strong decoupling and a high production of ROS, can activate energetic radical cascades and ROS-mediated cellular death. On the other hand substances that, also at the end of respiratory complexes, force the cellular respiration, such as dichloroacetate, thiamine or α-lipoic acid, induce ROS-mediated cell death in neoplastic cells and neuroprotection in many neurodegenerative diseases.

However in this context a special and interesting role is played by MLT, which exerts numerous physiological actions, through the modulation of various hormones, through actions carried through membrane and nuclear receptors. And probably by a modulation of numerous ion channels, but more distinctly, it affects the activity of many respiratory complexes, stimulating their functions and efficiency of electron transport. Last but not least, this molecule is converted *in vivo* into various metabolites, first of all the 6-OHM, and many others that could have a marked and increased activity modulating the respiratory complexes and particularly complex III.

Thus it is realized a real integrated system of biotransformation of indole compounds. In this context it is clear that in the mitochondrion, in the processes of cellular respiration, there is a common factor in oncologic and neurodegenerative pathologies.

The mitochondrion plays an essential function related to the synthesis of ATP stocks, has an important role in the homeostasis of calcium and potassium, and it plays a major role in the regulation of cellular death processes, through the intrinsic pathway of apoptosis.

A valuable contribution to studies on the origin of mitochondria and their role in the evolution was made by Lynn Margulis, who in the early 1970s published the essay “Origin of Eukaryotic Cells” in which the endosymbiotic hypothesis, in particular for mitochondria and chloroplasts, was revisited and proposed again [232]. The historical work written by Margulis can be expressed, even approximately, by the aphorism: “Life does not colonize life, but it is realized by means of interconnection and cooperation” so that, beyond many aspects still to be clarified, we can conclude that the main core of the endosymbiotic theory today appears highly probable and, for many profiles, attested [233].

From a strictly evolutionary viewpoint, the endosymbiosis between archaebacteria and proto-eukaryotes, which led to the development of the existing mitochondria, is undoubtedly an essential step for the development of life. It is clear how these organelles, in addition to the energy question, have a key role in the management of aerobe life and how the mitochondrion can adjust pO_2_ and in a sense functioning as a rheostat of oxygen tension, interfering on all signaling connected to it, such as those ROS mediated [113,114,115,116,117,118].

If in a strictly phylogenetic and evolutionary sense this cooperation led to the present design of life, today, more than ever, in the light of the literature and of above, we can conclude that the proper development and the proper differentiation of adult stem cells and the maintenance of a proper differentiated state, like the known genetic factors, depend on the relationship between pO_2_, mitochondrial function and metabolomics. Also, multiple aspects associated with cellular death and neurodegeneration depend on the respiratory function, regardless of phosphorylation.

In our opinion it is in this context and in this complex scenario that the role of MLT should be framed, considering it even in a strictly evolutionary sense; moreover, a prominent mechanism of action of the other molecules described above should be explained as the result of the action of those molecules on the mitochondrial respiratory complexes.

## Figures and Tables

**Table 1:** Values of the heat of formation and the self-consistent field energy for MLT, 6-OHM and 5,6-DHT.

Molecule	MLT	6-OHM	5,6-DHT
Heat of formation	−31.8208 kcal/mol	−77.0263 kcal/mol	−28.3233 kcal/mol
self-consistent field energy	−67,768.8155 kcal/mol	−75,162.8503 kcal/mol	−57,634.9772 kcal/mol

**Table 2:** Some properties of the molecules. The data are taken from ADC/Chemsketch log P plugin (Advanced Chemistry Development, Inc., Toronto, Canada) and The PubChem Project. USA: National Center for Biotechnology Information.

Molecule	Serotonin	*N*-Acetylserotonin	MLT	6-OHM	5,6-DHT
XLogP3	0.2	1	1.4	0.85	−0.1
Hydrogen bond donor count	2	3	2	3	4
Hydrogen bond acceptor count	2	2	2	3	3
